# Virulence characterization and clonal analysis of uropathogenic *Escherichia coli* metallo-beta-lactamase-producing isolates

**DOI:** 10.1186/s12941-021-00457-4

**Published:** 2021-08-03

**Authors:** Fatemeh Zangane Matin, Seyedeh Elham Rezatofighi, Mohammad Roayaei Ardakani, Mohammad Reza Akhoond, Fahimeh Mahmoodi

**Affiliations:** 1 Department of Biology, Faculty of Science, Shahid Chamran University of Ahvaz, 6135743135 Ahvaz, Iran; 2 Mathematical Sciences and Computer Faculty, Shahid Chamran University of Ahvaz, Ahvaz, Iran

**Keywords:** Uropathogenic *Escherichia coli*, Urinary tract infection, Metallo-beta-lactamase, Carbapenemase, Carbapenem resistance, Antimicrobial resistance, Virulence, *Bla*_NDM_, *Bla*_OXA-48_

## Abstract

**Background:**

Uropathogenic *Escherichia coli* (UPEC) is a major cause of urinary tract infection (UTI); however, treatment of UTI has been challenging due to increased antimicrobial resistance (AMR). One of the most important types of AMR is carbapenem resistance (CR). CR bacteria are known as an important threat to global public health today. Class B metallo-beta-lactamases (MBLs) are one of the major factors for resistance against carbapenems. We aimed to investigate the characteristics of UPEC isolates producing MBL.

**Methods:**

A cross-sectional study was conducted from October 2018 to December 2019 in Ahvaz; Iran. UPEC isolates were identified by biochemical and molecular methods. Metallo-beta-lactamase-producing isolates were detected using modified carbapenem inactivation method (mCIM) and EDTA-CIM (eCIM) tests. MBL genes, phylogenetic group, and virulence genes profile of carbapenem resistant isolates were determined. Conjugation assay and plasmid profiling were conducted to evaluate the ability of transferring of CR to other *E. coli* isolates. Clonal similarity of isolates were assessed using *Enterobacterial* intergenic repetitive element sequence (ERIC)-PCR.

**Results:**

Among 406 UPEC isolates, 12 (2.95%) carbapenem-resistant were detected of which 11 were phenotypically MBL-producing strains. Four isolates were resistant to all investigated antimicrobial agents and were considered possible pandrug-resistant (PDR). *bla*_NDM_, *bla*_OXA-48_, *bla*_IMP-1_, and *bla*_IMP-2_ genes were found in 9, 5, 1, and 1 isolates, respectively. Among 30 virulence genes investigated, the *traT, fyuA* followed by *fimH*, and *iutA* with the frequency of 8 (66.7%), 8 (66.7%), 7 (58.3%), and 7 (58.3%) were the most identified genes, respectively. Siderophore production was the main virulence trait among carbapenem-resistant UPEC isolates. Except for two, all other isolates showed weak to moderate virulence index. In all recovered isolates, CR was readily transmitted via plasmids to other isolates during conjugation experiments.

**Conclusion:**

MBL and carbapenemase genes, especially *bla*_NDM_ and *bla*_OXA-48_ are spreading rapidly among bacteria, which can be a threat to global public health. Therefore monitoring the emergence and dissemination of new AMR is necessary to continuously refine guidelines for empiric antimicrobial therapy. Understanding the mechanisms of resistance and virulence in this group of bacteria can play an effective role in providing new therapeutic methods.

## Introduction

Urinary tract infection (UTI) is one of the most common infectious diseases that affects people of all ages. The main etiologic agent causing UTI is a group of *Escherichia coli* strains that named uropathogenic *Escherichia coli* (UPEC) [1]*.* UPEC is a subgroup of extraintestinal pathogenic *E. coli* (ExPEC) which is differentiated from intestinal *E. coli* strains. UPEC strains are capable to colonize and invade urogenital tract which eventually leads to infection of urinary tract system. Disease establishment is caused by virulence factors (VFs) of bacteria and host characteristics [2, 3]. Various VFs have been incriminated in UPEC pathogenesis including invasins, adhesins, toxins, iron-acquisition systems, and serum resistance factors [4]. VFs may be present in the chromosome or acquired horizontally through mobile genetic elements such as transposons, plasmids, and pathogenicity islands, thereby leading to great diversity among UPEC strains [5, 6].

Antimicrobial therapy usually is the first strategy in treating a UTI. The choice of antimicrobial compound depends on the patient's health condition and the type of bacteria causing the UTI. However, due to misuse of antibiotics, antimicrobial resistance (AMR) has greatly expanded among bacteria. Many *E. coli* strains, especially UPEC have become multi-, extensively- or pan-drug resistant (MDR, XDR, or PDR) [7, 8]. This poses a great challenge in the treatment of UTI infections. Therefore, monitoring the emergence and dissemination of new AMR is necessary to continuously refine guidelines for empiric antimicrobial therapy [1, 9]. Resistance against trimethoprim-sulfamethoxazole, fluoroquinolones, and beta-lactams is increasing among UPEC isolates [2, 10, 11]. Carbapenems such as imipenem, meropenem, and doripenem are stable antibiotics against extended-spectrum β-lactamases (ESBLs) and AmpC β-lactamases [12] and considered as a last-resort drugs for treating infections by MDR bacteria. However extensive use of carbapenems has led to the emergence of carbapenem-resistant *Enterobacteriaceae* (CRE) [12]. CRE are known as a global clinical and public health problem because their infections are resistant to most classes of antibiotics including trimethoprim-sulfamethoxazole, fluoroquinolones, and beta-lactams and so-called superbugs. CRE are associated with high mortality due to limited treatment options [12–14].

Several mechanisms have been proposed for resistance against carbapenems in *Enterobacteriaceae*. Production of carbapenemases that are categorized in Ambler classification as follows: class A carbapenemases including *Klebsiella pneumonia* carbapenemase (KPC), Guiana extended-spectrum β-lactamase (GES), Serratia marcescens enzyme (SME), imipenemase/non-metallocarbapenemase-A (IMI/NMC-A), sulfhydryl variable lactamase (SHV), and Serratia fonticola carbapenemase (SFC-1); class B metallo-beta-lactamase (MBL) including New Delhi metallo-beta-lactamase (NDM), imipenemase (IMP), and Verona integron-encoded metallo-beta-lactamase (VIM), as well as class D oxacillinases (OXA). Moreover, over-expression of ESBLs, AmpC enzymes and efflux pumps combined with porin loss can also lead to carbapenem resistance (CR) [12, 15–18]. Carbapenemase genes are predominantly located on plasmids and are transmitted to other bacteria or integrated into chromosomes [16].

In the present study we aimed to investigate the antimicrobial susceptibility pattern and presence of MBL-producing among UPEC isolates. As the information on the virulence characteristics of MBL-producing UPEC isolates is very limited, the virulome, phylogenetic groups, clonal relationship, and mechanism of CR transfer among these isolates were investigated.

## Materials and methods

### Ethics

The ethics of the study was confirmed by the Ethics Committee of Shahid Chamran University of Ahvaz according to Declaration of Helsinki (EE/98.24.3.26336/scu.ac.ir). Before collecting information, participants or parents (for children cases) were asked to read, accept and sign an informed consent form.

### Sample collection and identification

A cross-sectional study was performed from October 2018 to December 2019. The sample size was estimated using a single population proportion formula based on the prevalence of 0.15 [1], 95% confidence interval, and margin error of 5%. With considering a 10% non-response rate, the minimum samples size was 225; however, we collected 427 *E. coli* isolates for more accuracy.

The suspected *E. coli* isolates were obtained from hospitals and laboratories in Ahvaz city; Iran. These bacteria were isolated from patients who suffered from UTI and referred to laboratories by physicians to identify the pathogen and performing the antibiogram test. UTI was defined as the presence of at least 10^5^ cfu/mL of pathogenic agent in urine and pyuria (≥ 10^4^ leukocytes/mL of urine). The midstream urine samples of these patients were cultured on Blood Agar and McConkey and after purification, they were examined for antibiotic susceptibility profile. Thus, 427 isolated *Escherichia coli* isolates were collected and further investigated. *E. coli* isolates of patients who had recently consumed antimicrobial drugs were excluded from the study. Briefly, to identify and confirm the isolates, they were cultured onto MacConkey (Biolife Italiana; Italy) and Eosin Methylene Blue (EMB, Merck; Germany), and subsequently were incubated at 37 °C for 24 h. Lactose-fermenting colonies on MacConkey, or colonies with metallic sheen on EMB were investigated by conventional biochemical tests including production of lysine decarboxylase, oxidase, Sulfur Indole Motility (SIM), Simmon’s Citrate and Methyl Red/Voges-Proskauer (MR/VP). Finally, purified isolates were analyzed by PCR for the presence of *uspA* gene, which is the highly specific gene of *E. coli*. Detection of *uspA* gene was performed as described previously [19].

### Antimicrobial susceptibility pattern

Kirby-Bauer disc diffusion method was performed to evaluate the resistance and susceptibility of isolates to antimicrobial agents recommended by Clinical & Laboratory Standards Institute 2018 (CLSI-2018) [20]. The antimicrobial discs included Nalidixic-acid, Ampicillin, Tetracycline, Streptomycin, Sulfamethoxazole-trimethoprim, Ciprofloxacin, Kanamycin, Gentamycin, Fosfomycin, Imipenem, Meropenem, Cefotaxime, Ceftazidime, Cefazolin, and Nitrofurantoin. The isolates resistance to three or more different antimicrobial families were considered multidrug resistant (MDR).

As meropenem or imipenem resistant UPEC isolates were the focus of present study, the resistant isolates were subjected to further analyses. The minimum inhibitory concentration (MICs) for imipenem or meropenem were determined as recommended by CLSI-2018. MIC breakpoints for both antibiotics were defined ≥ 4 μg/mL. The resistance of these isolates to ceftriaxone, cefoxitin, cefepime, amikacin, ampicillin-sulbactam, aztreonam, piperacillin/tazobactam, and colistin antimicrobials was also measured to determine XDR and PDR isolates. The CLSI recommendation for *Acinetobacter* spp. was applied for colistin.

### Combined-disc (CD) and double disc synergy (DDS) tests

Phenotypic CD and DDS tests were performed to identify MBL-producing isolates. For CD test, two discs of imipenem (10 µg) and imipenem-EDTA (10 µg-1460 µg) were placed on the Muller-Hinton agar (MHA; Biolife Italiana; Italy) inoculated by 0.5-McFarland test isolates. After incubation of the plates at 35 °C for 16–18 h, the inhibition zone around discs were measured. The isolates were considered MBL-producer when the diameter of inhibition zone around the imipenem-EDTA disc increased by ≥ 7 mm compared to imipenem disc alone [21].

For DDS test, two discs of imipenem and EDTA (1460 µg) were placed on MHA plates inoculated with test isolates. The distance between two discs was considered 15 mm. After incubation of the plates at 35 °C for 16–18 h, the zone around discs were measured. Increasing of the inhibition zone or the formation of a phantom zone between the two discs indicated the MBL production [21].

### Modified carbapenem inactivation method (mCIM) and EDTA-CIM (eCIM)

Either mCIM and eCIM tests are extensively used for the epidemiological or infection prevention aims. The mCIM test could identify the bacteria that produce all carbapenemases, while the eCIM test was performed to differentiate MBL-producers from the serine carbapenemases. To perform mCIM, 1 µL loopful of the isolates were emulsified in 2 mL of tryptone soya broth (TSB). Then, one meropenem disc was immersed in the suspension for 4 h at 37 °C. A MHA plate was inoculated by 0.5-McFarland standard *E. coli* ATCC25922. Meropenem disc was removed from the suspension and excess liquid was expelled. Meropenem disc was placed on the inoculated plate and incubated at 37 °C for 24 h. Inhibition zone diameter of 6–15 mm or appearance of pinpoint colonies within a 16–18 mm zone around imipenem disc indicate the presence of carbapenemase [20].

eCIM test was performed when the mCIM test was positive. This test was done as similar to mCIM, except that after adding test isolate to the TSB, 20 µL of 0.5 M EDTA was added; then meropenem disc was immersed. Meropenem discs of eCIM and mCIM tests were placed on one plate and analyzed simultaneously. An increase of ≥ 5 mm in inhibition zone for eCIM versus mCIM was considered MBL-positive, while no change in zone diameter or an increase of ≤ 4 mm indicated the presence of carbapenemase [20].

### Phenotypic differentiation of MLBs and class A KPC carbapenemases

To differentiate MBLs- and class A KPC carbapenemase-producing isolates, phenyl boronic acid (PBA) disc test was applied. The test was performed as a combined-disc of meropenem with and without PBA. Phenyl boronic acid was dissolved in dimethyl sulfoxide (DMSO) at a concentration of 20 mg/mL. Then meropenem disc was inoculated by 20 µL of PBA solution (400 µg PBA/disc). The test was performed as given for the standard disc diffusion method. An increase of ≥ 5 mm in inhibition zone around meropenem-PBA disc versus meropenem was considered class A KPC carbapenemase producer [22, 23].

### Detection of resistance genes

Imipenem or meropenem-resistant isolates were analyzed for the presence of MBL-genes including *bla*_VIM-1_, *bla*_VIM-2_, *bla*_IMP-1_, *bla*_IMP-2_, *bla*_SPM-1_, *bla*_NDM_, *bla*_SIM_, and *bla*_GIM_. The other CR genes of *bla*_KPC_, *bla*_OXA-23_ and *bla*_OXA-48_ were also investigated. Genomic DNA of isolates was extracted using the boiling lysis procedure. PCR reactions were performed as previously described [24–31].

### Virulence genotyping and phylogenetic grouping

The isolates were assayed for the presence of 30 virulence traits using five multiplex-PCR panel as previously described [32]. The investigated VGs were including *papEF*, *papA*, *fimH*, *papG allele I-III*, *papG allele I*, *papG allele II*, *papG allele III*, *kspMTIII*, *gafD*, *focG*, *sfa/focDE*, *nfaE*, *papC*, *afa/draBC*, *sfaS*, *bmaE*, *ibeA*, *traT*, *cvaC*, *cdtB*, *hlyA*, *cnf1*, *fyuA*, *chuA*, *iutA*, *K1*, *K5*, *kpsMTII*, and *rfc*. Determination of major *E. coli* phylogroups was performed based on the quadruplex PCR, and complementary tests as described by Clermont et al. [33]. According to the presence of three genes of *arpA*, *chuA* and *yjaA*, and TSPE4.C2 DNA fragment, the isolates assigned to one of A, B1, B2, C, D, E and F phylogenetic groups.

### Enterobacterial intergenic repetitive element sequence (ERIC)-PCR

Fingerprinting of imipenem or meropenem resistant isolates was performed using ERIC-PCR based on conditions and primers described previously [34]. To evaluate the relationship between isolates, the presence or absence of bands compared to the standard DNA molecular marker was assessed. The clustering of the isolates was done based on Unweighted Pair Group Method with Arithmetic Mean (UPGMA) analysis using the SAHN NTSYS program version 2.02e. A Dice similarity index was used for the definition of ERIC clusters.

### Plasmid profiling and conjugation

Carbapenem resistant isolates were assayed for plasmid content. Plasmids were extracted using alkaline lysis method [35], then electrophoresed on agarose gel (1%). Estimation of plasmid sizes was acquired using a molecular weight marker, made from a lambda/*Hind* III digest.

The MBLs- or carbapenemase-producing isolates were conjugated with a lactose-negative enteroinvasive *E. coli* (EIEC) strain that was susceptible to imipenem and meropenem. The donors and recipient bacteria were cultured in nutrient broth for 16 h; then mixed in a ratio of 1:10 (donor: recipient) [35]. After incubation for 48 h at 37 °C, the mixtures were inoculated on MCA containing imipenem or meropenem (4 μg/mL). The plates were incubated overnight at 37 °C. The resistant lactose-negative isolates were analyzed for the presence of *inv* and resistance genes via PCR reaction.

## Results

### Antimicrobial susceptibility pattern of UPEC isolates

Out of 427 cultured samples, 406 isolates were phenotypically and molecularly confirmed as UPEC. As defined in Table 1, the most antimicrobial resistance was against ampicillin (82.3%) followed by cefazolin (80%), tetracycline (59.4%), and nalidixic-acid (59.1%). Only 13 (3.2%) isolates were susceptible to all antimicrobials tested and the rest (96.8%) were resistant to at least one or more antimicrobials. MDR profile was found in 377 (92%) isolates. The highest frequency of antimicrobial susceptibility was recorded for meropenem (97.5%), imipenem (97.3%), followed by nitrofurantoin (95.4%), and fosfomycin (92.4%).

**Table 1:** Antimicrobial resistance profile of all uropathogenic *Escherichia coli* and carbapenem-resistant isolates

Category of antibiotic		Quinolone	Penicillin	Tetracycline	Streptomycin	Folate pathway inhibitors	Fluoroquinolone	Aminoglycoside	Aminoglycoside	Phosphonic acid	Carbapenem	Carbapenem
Antibiotic		NA N (%)	AMP N (%)	TET N (%)	STR N (%)	SXT N (%)	CP N (%)	K N (%)	GM N (%)	FOS N (%)	IMP N (%)	MEP N (%)
UPEC isolates (n = 406)	Resistant	240 (59.1)	334 (82.3)	241 (59.4)	175 (43.1)	222 (54.7)	181 (44.6)	93 (22.9)	88 (21.7)	31 (7.6)	11 (2.7)	10 (2.5)
	Intermediate	51 (12.6)	18 (4.5)	11 (2.7)	130 (32)	11 (2.7)	16 (4.0)	132 (32.5)	10 (2.5)	25 (6.2)	0 (0)	0 (0)
Carbapenem-resistant isolates (n = 12)	Resistant	12 (100)	12 (100)	10 (83.3)	10 (83.3)	11 (91.6)	12 (100)	11 (91.6)	11 (91.6)	8 (66.6)	11 (91.6)	10 (83.3)
	Intermediate	0 (0)	0 (0)	1 (8.3)	0 (0)	0 (0)	0 (0)	1 (8.3)	1 (8.3)	1 (8.3)	0 (0)	0 (0)

Cephalosporin	Cephalosporin	Cephalosporin	Nitrofuran	Cephalosporin	Cephamycin	Extended-spectrum cephalosporins	Aminoglycosides	Penicillins + β-lactam Inhibitor	Monobactams	Penicillins + β-lactam Inhibitor	Polymyxin
CTX N (%)	CAZ N (%)	CZ N (%)	FM N (%)	CRO N (%)	FOX N (%)	FEP N (%)	AN N (%)	SAM N (%)	AZT N (%)	PTZ N (%)	CL N (%)
222 (54.7)	176 (43.4)	325 (80)	15 (4.6)	–	–	–	–	–	–	–	–
28 (6.9)	40 (9.9)	59 (14.5)	9 (2.2)	–	–	–	–	–	–	–	–
12 (100)	11 (91.6)	12 (100)	5 (41.6)	10 (100)	10 (83.3)	12 (100)	11 (91.6)	12 (100)	12 (100)	10 (83.3)	11 (91.6)
0 (0)	0 (0)	0 (0)	1 (8.3)	0 (0)	0 (0)	0 (0)	0 (0)	0 (0)	0 (0)	1 (8.3)	0 (0)

*UPEC* uropathogenic *Escherichia coli*, *NA *nalidixic-acid, *AMP* ampicillin, *TET* tetracycline, *STR* streptomycin, *STX* Sulfamethoxazole-trimethoprim, *CP* ciprofloxacin, *K* kanamycin, *GN* gentamycin, *FOS* fosfomycin, *IMP* Imipenem, *MEN* meropenem, *CTX* cefotaxime, *CAZ* ceftazidime, *CZ* cefazolin, *FM* nitrofurantoin, *CRO* Ceftriaxone, *FOX* cefoxitin, *FEP* cefepime, *AN* Amikacin, *SAM* ampicillin-sulbactam, *AZT* aztreonam, *PTZ* piperacillin/tazobactam, *CL* colistin

In total, 12 isolates were resistant to imipenem, meropenem or both (Table 1) and archived for further analysis. Five isolates were XDR meaning non-susceptible to at least one drug in all but two or fewer antibiotic or antimicrobial family [8], also 4 (1%) isolates were possible PDR meaning were non-susceptible to all investigated antimicrobial categories [8].

### Phenotypic detection of MBL-producing UPEC isolates

**Fig. 1:**
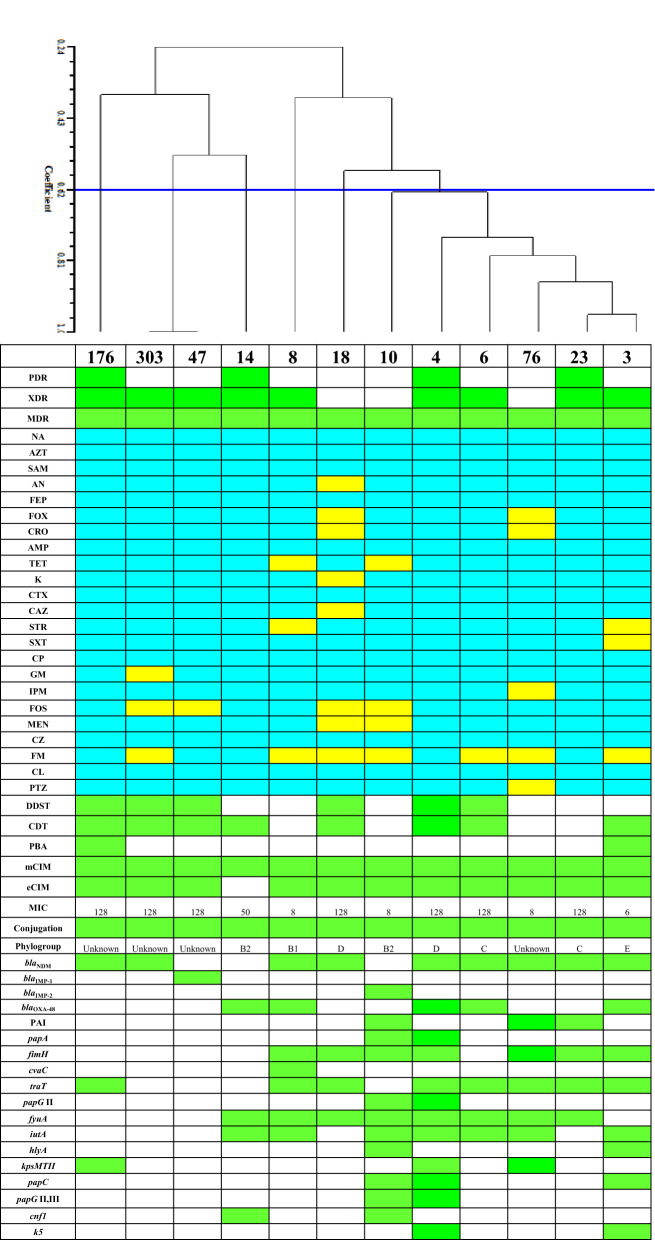
ERIC-PCR clustering and all phenotypic and genotypic characters of carbapenem-resistant UPEC isolates. *bla*_VIM-1_, *bla*_VIM-2_, *bla*_SIM_, *bla*_SPM_, *bla*_GIM_, *bla*_OXA-23_, *bla*_KPC_, *kpsMTIII*, *papEF*, *ibeA*, *gafD*, *cdtB*, *focG*, *bmaE*, *sfa*/*focDE*, *papG* allel I, II, and III, *K1*, *rfc*, *nfaE*, *sfaS*, and *afa/draBC* genes were not found; therefore were not shown. Blue and yellow colors indicate resistance and susceptibility, respectively. Green color shows presence of gene or phenotypic character and white color indicates absence of gene or phenotypic character. Abbreviations were as follow: *PDR *pan-drug resistant, *XDR* extensively-drug resistant, *MDR* multi-drug resistant, *NA* nalidixic-acid, *AMP* ampicillin; *TET* tetracycline, *STR* streptomycin, *STX *sulfamethoxazole-trimethoprim, *CP* ciprofloxacin, *K* kanamycin, *GN* gentamycin, *FOS* fosfomycin, *IMP* imipenem, *MEN *meropenem, *CTX *cefotaxime, *CAZ* ceftazidime, *CZ *cefazolin, *FM *nitrofurantoin, *CRO* ceftriaxone, *FOX *cefoxitin, *FEP* cefepime, *AN* amikacin, *SAM* ampicillin-sulbactam, *AZT* aztreonam, *PTZ* piperacillin/tazobactam, *CL *colistin, *DDST* double disc synergy test, *CDT* combined disc test, *PBA* phenylbronic acid disc test, *mCIM* modified carbapenem inactivation method, *eCIM* EDTA-CIM, *MIC *minimum inhibitory concentration

In total, 9 isolates were confirmed by disc diffusion and MIC to be resistant against imipenem and meropenem, however, 2 and 1 isolates were only resistant against imipenem and meropenem, respectively. To detect MBL-producers, CDT, DDST, and mCIM-eCIM were done. We found that 2 and 6 UPEC isolates were positive for CD, or both CD, DDS tests, respectively; while four UPEC isolates were negative in both tests (Table 2; Fig. 1).

**Table 2:** Phenotypic and genotypic results of cabapenem-resistant uropathogeneic *Escherichia coli* isolates

Test	DDST N (%)	CDT N (%)	PBA N (%)	mCIM N (%)	eCIM N (%)	*bla*_NDM_ N (%)	*bla*_IMP-1_ N (%)	*bla*_IMP-2_ N (%)	*bla*_VIM-1_ N (%)	*bla*_VIM-2_ N (%)	*bla*_SIM_ N (%)	*bla*_GIM_ N (%)	*bla*_SPM-1_ N (%)	*bla*_kpc_ N (%)	*bla*_oxa-23_ N (%)	*bla*_oxa-48_ N (%)
Sum	6 (50)	8(66.7)	2(16.7)	12 (100)	11(91.6)	9 (75)	1 (8.3)	1 (8.3)	0 (0)	0 (0)	0 (0)	0 (0)	0 (0)	0 (0)	0 (0)	5 (41.7)

*DDST* double disc synergy test, *CDT* combined disc test, *PBA* phenylbronic acid disc test, *mCIM* modified carbapenem inactivation method, *eCIM* EDTA-CIM

Phenotypic tests of mCIM, eCIM were used to detect and differentiate MBLs from serine carbapenemases. Out of 12 resistant UPEC isolates, 11 were positive by mCIM-eCIM tests, while one isolate was negative by eCIM. Details of results are presented in Table 2 and Fig. 1.

### Phenotypic detection of KPC-producing UPEC isolates

PBA disc test was applied to detect class A KPC-producing isolates. Two isolates were phenotypically positive by this test and were considered KPC-producing UPEC. However, these isolates were also previously detected as MBL producers (Table 2; Fig. 1).

### Resistance gene assays of MBL- and carbapenemase-producing isolates

The distribution of MBLs and carbapenemase genes among UPEC isolates are shown in Table 2. Out of 12 carbapenem resistant isolates, 11 were positive for MBL genes. *bla*_NDM_, *bla*_IMP-1_, and *bla*_IMP-2_ were found in 9, 1, and 1 isolates, respectively. None of the isolates carried *bla*_VIM-1_, *bla*_VIM-2_, *bla*_SIM_, *bla*_SPM_, and *bla*_GIM_ genes. Three main non-MBL carbapenemase genes of *bla*_OXA-48_, *bla*_OXA-23_, and *bla*_KPC_ were investigated [36–40]. *bla*_OXA-48_ was found in five isolates while *bla*_OXA-23_, and *bla*_KPC_ were not detected. The simultaneous carriage of two genes *bla*NDM and *bla*OXA-48 was found in four isolates (Table 4; Fig. 1). Gene sequences were registered under the accession numbers of MT321108 (*bla*_NDM_), MT321107 (*bla*_NDM_), MT321106 (*bla*_OXA-48_), and MT293867 (*bla*_IMP_) in the GenBank.

### Virulome and phylogenetic grouping

The content of VGs for carbapenem resistant UPEC isolates is shown in Table 3. The most detected genes were *traT, fyuA* followed by *fimH*, and *iutA* with the frequency of 8 (66.7%), 8 (66.7%), 7 (58.3%), and 7 (58.3%), respectively. *kpsMTIII*, *papEF*, *ibeA*, *gafD*, *cdtB*, *focG*, *bmaE*, *sfa*/*focDE*, *papG* allel I, II, and III, *K1*, *rfc*, *nfaE*, *sfaS*, and *afa/draBC* genes were not detected among the isolates. Two UPEC isolates had none of the tested virulence genes while they showed resistance against all investigated antibiotics. According to phylogenetic grouping 2, 2, 2, 1, and 1 isolates were B2, C, D, B1, and E phylogroups, respectively. Four isolates were positive for all three genes and DNA fragment and according to Clermont *Escherichia coli* phylo-typing were considered as “unknown” [33].

**Table 3:** Frequency of virulence genes among carbapenem-resistant uropathogeneic *Escherichia coli* isolates

Gene	N (%)	Group of virulence trait	Number of detected genes N (%)	Number of isolates with at least one virulence gene N (%)
*papEF*	0 (0)			
*papA*	2 (16.7)			
*fimH*	7 (58.3)			
*papG allele I-III*	2 (16.7)			
*papG allele I*	0 (0)			
*papG allele II*	2 (16.7)			
*papG allele III*	0 (0)	Adhesions	16 (7.8%)	8 (66.7)
*kspMTIII*	0 (0)			
*gafD*	0 (0)			
*focG*	0 (0)			
*sfa/focDE*	0 (0)			
*nfaE*	0 (0)			
*papC*	3 (25)			
*afa/draBC*	0 (0)			
*sfaS*	0 (0)			
*bmaE*	0 (0)			
*ibeA*	0 (0)	Invasion	0 (0)	0 (0)
*traT*	8 (66.7)	Serum resistance	9 (37.5)	8 (66.7)
*cvaC*	1 (8.3)			
*cdtB*	0 (0)			
*hlyA*	2 (16.7)	Toxin	4 (11.1)	3 (25)
*cnf1*	2 (16.7)			
*fyuA*	8 (66.7)			
*chuA*	6 (50)	Siderophore	20 (55.5)	9 (75)
*iutA*	7 (58.3)			
*K 1*	0 (0)			
*K 5*	2 (16.7)	Protectin	5 (10.4)	3 (25)
*kpsMTII*	3 (25)			
*rfc*	0 (0)			
PAI	3 (25)	Pathogenic island	3 (25)	3 (25)
unknown	4 (33.3)			
B1	1 (8.3)			
B2	2 (16.7)	Phylogroup		
D	2 (16.7)			
C	2(16.7)			
E	1 (8.3)			

### ERIC-PCR

The isolates produced PCR products with different sizes from 250 to 2500 bp and various patterns yielded 5–12 bands. Two isolates showed the same ERIC pattern and virulence gene profile; however the AMR patterns were different. Also two isolates showed ~ 95% similarity index. The similarity of the other isolates was < 80%, indicating a different source of isolates. Drawn dendrogram had a matrix correlation of 0.91.

### Conjugal transfer and plasmid profiling

Conjugation test showed that imipenem or meropenem resistance could be transmitted to other bacteria; therefore, these isolates were capable to disseminate MBL or carbapenemase genes horizontally. All 12 UPEC donors transferred carbapenem resistance phenotype to EIEC recipient strain and this strain was able to grow on the medium containing the antibiotic carbapenem. Transconjugants were positive for MBL or carbapenemase genes and *inv* by gene-specific PCR. Plasmid profiling showed different patterns for plasmids ranging 5 to upper 50 kb. All isolates presented different plasmid pattern. Plasmid profiling of donors and recipients cells were not equal after conjugation, indicating that not all plasmids were transferred during conjugation.

## Discussion

UTI is one of the most important bacterial infectious diseases in humans, which is mainly caused by *E. coli*. Antibiotics are commonly used to treat UTIs. However, the emergence and dissemination of AMR has posed a major challenge in the treatment of these diseases. Detection of antibiotic resistance type, its transmission mechanism, and the characteristics of resistant bacteria can be very effective in designing treatment guidelines.

Investigation of antimicrobial susceptibility profile of UPEC isolates shows high antimicrobial resistance against most of the antibiotics evaluated in this study. However, imipenem, meropenem, nitrofurantoin, fosfomycin, and to some extent gentamycin and kanamycin showed noticeable activity against UPEC isolate. Developing resistance to these antibiotics can be very costly to the healthcare system. Other studies in Iran regarding the antibiotic resistance of UPEC isolates show almost similar profiles [42–44]. Some studies in Iran have not found resistance to imipenem or meropenem among *E. coli* isolates. More than 90% of UPEC isolates were MDR and 2.2% were possible XDR. This statistic indicates a high frequency of antibiotic resistance among these isolates. Four isolates were resistant to all studied antibiotics and therefore were considered possible PDR. The XDR and PDR *Enterobacteriaceae* are important because the mortality rate is high among patients infected by these bacteria [46–47]; although, some researchers disagree [48]. PDR *E. coli* is very rare [45]; however, in our study, four isolates (1%) were resistant to all the antibiotics studied. Risk factors for infections with XDR and PDR *E. coli* isolates have not yet been identified and little information is available. Understanding the mechanisms of AMR and virulence characteristics of these pathogens helps researchers to find solutions for the long-standing AMR problem [7].

To detect MBL-producing isolates and differentiate from other carbapenemases CDT, DDST, mCIM-eCIM and PBA test were performed. Previously, Modified Hodge test was recommended as a phenotypic method for detecting carbapenemases; however, this test was excluded from the CLSI (2018) due to the inability to identify some carbapenemase-producing bacteria, including NDM-producing strains [21]. Among the tests performed in the present study, the mCIM-eCIM method was able to more effectively detect the MBL-producing isolates. MBL genes were found in all isolates identified as MBL-producers by this method. One isolate was positive by mCIM but negative by eCIM and also PBA disc test. Eventually it was found that this isolate carries *bla*_OXA-48_ and belonged to group D carbapenemases. Although two isolates were positive for PBA disc test, but they lacked *bla*_KPC_ gene. These isolates were also positive for mCIM-eCIM and both had the *bla*_NDM_ gene. The lack of detection of *bla*_KPC_ may be due to the inability of the primers to target this gene or the PBA disc test results were false positive.

PCR detection and sequencing showed that 11 isolates carry MBL genes. The predominant gene was *bla*_NDM_ that was found in nine UPEC isolates. For the first time *bla*_NDM_ was detected in a Swedish patient (originally from India) who traveled to New Delhi, India. In this patient, the *bla*_NDM_ was isolated from *Klebsiella pneumoniae*, which caused UTI [49]. After that, bacteria carrying *bla*_NDM_ have been reported from other parts of the world including Iran [51–53]. In Iran *bla*_NDM_ is mostly isolated from carbapenem-resistant *Klebsiella* isolates [54, 55]; however, there have been reports of carbapenem-resistant *E. coli* isolates carrying this gene [53, 57–58]. A study conducted in Ahvaz; Iran from 2014 to 2015 reported that there were no MBL-producing *Enterobactericeae* isolates carrying *bla*_NDM_ in this region [59]. However, in 2018, this gene was found in commensal *E. coli* and *Pseudomonas aeruginosa* which indicates the dissemination of this type of resistance among bacteria in Ahvaz; Iran [57, 60]. In the present study, 2.2% of UPEC isolates harbored *bla*_NMD_; therefore, this gene is rapidly spreading among the strains of this region. Conjugation experiments showed that all *bla*_NDM_ genes identified in this study are transferred by plasmids and have the ability to be transmitted to other strains. The other detected MBL genes were *bla*_IMP-1_ and *bla*_IMP-2_. Little is known about the presence of these genes among carbapenem-resistant *Escherichia coli* isolates in Iran. We found two reports about the presence of *bla*_IMP_ among *E. coli* strains in northern Iran and Tehran (capital of Iran) [61, 62]. *bla*_OXA-48_, which is class D carbapenemase, was detected in five carbapenem-resistant UPEC isolates. This gene also seems to play an important role in carbapenem resistance among *E. coli* isolates. *bla*_OXA-48_ is endemic in Iran and dissemination of this gene is mainly driven by the composite transposon Tn1999 and its variants [63].

In the present study, the co-existence of *bla*_OXA-48_ and *bla*_NDM_ genes was found in four UPEC isolates. However, it is not clear that these two genes are carried on a single plasmid or placed in the separate plasmids. The presence of these two genes in an isolate has been reported in several studies [15, 17, 63].

Conjugation experiments revealed that all carbapenem resistance genes were able to transfer to other isolates; however, plasmid profiling showed that these genes carried by plasmids with different sizes and probably different sequences. We could not identify the sequence and types of the plasmids which is another limitation of the present study. Complete sequencing of these plasmids can clear up many ambiguities about how they are transmitted, distributed, and their role in the resistance and virulence of pathogenic bacteria. ERIC-PCR clusters indicated that some isolates were clonally similar, but revealed different AMR profiles and different carbapenem- resistant genes that may be due to having different plasmids.

Little is known about the pathogenicity of carbapenem-resistant *E. coli* strains. Most virulence and antibiotic resistance genes, especially CR genes, are transmitted to bacteria via plasmids. However, the ability of bacteria to accept different plasmids is limited because plasmids have cost for bacteria and the energy resources are limited. It is not clear whether bacteria move toward greater resistance, greater pathogenicity, or both during their evolution. The finding of Gottig et al. revealed that NDM-1 carriage and expression exerted a fitness cost and does not significantly affect virulence [48]. Investigation of the presence of 30 virulence genes in carbapenem-resistant isolates showed that these isolates had from 0 to 10 virulence genes. No virulence gene were found in the isolates 303 and 47 (Table 4). Although they had different antibiotic resistance profile, ERIC-PCR clustering showed that they are the same clone. The isolate 4, which was considered possible PDR, had ten virulence genes; thus, this isolate had high potential virulence and antibiotic resistance at the same time. The isolate 10 also showed a strong virulence profile; however, the virulome of the rest of them reflected low virulence index.

**Table 4:** Virulence genes and antimicrobial resistance scores of carbapenem-resistant uropathogenic *Escherichia coli* isolates

Name	Score of VG (N = 30) Mean: 4.3	Score of AMR (N = 15) Mean: 13.25	Detected resistance genes
3	6	12	*bla*_NDM_-*bla*_OXA-48_
4	10	15	*bla*_NDM_-*bla*_OXA-48_
6	3	14	*bla*_NDM_-*bla*_OXA-48_
8	5	12	*bla*_NDM_-*bla*_OXA-48_
10	10	11	*bla*_IMP-2_
14	3	15	*bla*_OXA-48_
18	3	10	*bla*_NDM_
23	4	15	*bla*_NDM_
47	0	15	*bla*_IMP-1_
76	6	13	*bla*_NDM_
176	2	15	*bla*_NDM_
303	0	12	*bla*_NDM_

*VG* virulence gene, *AMR* antimicrobial resistance

The genes including *traT*, *fimH*, *fyuA*, and *iutA* were more prevalent among CR isolates. However, when the frequency of virulence genes was assessed based on their function, the genes involved in iron uptake and siderophore production (*fyuA*, *iutA*, and *chuA*) were highest and 75% of these isolates carried at least one of these genes. *fyuA* (yersiniabactin siderophore) also plays an important role in biofilm formation in urinary tract system and detaching host-derived copper, it thus protect against intracellular killing [64]. El Ghany et al. characterized 10 carbapenem resistant UPEC isolates. They found that although these isolates have different virulence structure; they nevertheless contained at least one iron system gene [2]. Serum resistance traits (*traT* and *cvaC* genes) was the other important virulence factor that was found among these isolates. Toxin, protectin, and adhesion virulence factors were of lower importance. Although 58% of the isolates had the *fimH* gene, the evaluation of the presence of another 15 adhesion genes revealed that the acquisition or maintenance of these genes was less important for these bacteria. In contrast to our results, Ranjan et al. reported that all five investigated NDM-harboring strains inherited genes belonging to adhesions and reflected moderate virulence [16].

Further studies are needed to determine what virulence factors exist in carbapenem-resistant bacteria after gaining resistance. Perhaps a new treatment can be found to overcome these pathogens by targeting common biochemical pathways for the synthesis of proteins related to the virulence and resistance genes.

## Conclusion

Antimicrobial resistance, especially resistance to carbapenems, is rapidly expanding. CR is easily transmitted to other bacteria through HGT. The *bla*_NDM_ followed by *bla*_OXA-48_ were the most common cause of producing metallo-beta-lactamase and carbapenemase among the carbapenem-resistant isolates. Virulome analysis of these isolates revealed the low virulence index in most of them; however, genes involved iron uptake genes especially siderophores were prevalent in the CR isolates. Understanding the spreading and pathogenicity in this group of emerging resistant bacteria can help to improve the therapeutic options and stewardship strategies in different regions.

## Data Availability

The datasets used and/or analyzed during the current study are available from the corresponding author on reasonable request. Sequence data of this project have been deposited in the GenBank of the National Center for Biotechnology Information (NCBI) under the accession number MT321108, MT321107, MT321106, and MT293867.

## Abbreviations

UPEC: Uropathogenic *Escherichia coli*
UTI: Urinary tract infection
ERIC: Enterobacterial intergenic repetitive element sequence
ExPEC: Extraintestinal pathogenic *E. coli*
VF: Virulence Factor
UPGMA: Unweighted pair group method with arithmetic mean
TSB: Trypton soya broth
AMR: Antimicrobial resistance
MDR: Multi-drug resistant
XDR: Extensively-drug resistant
PDR: Pan-drug resistant
CR: Carbapenem resistance
MBLs: Metallo-β-lactamases
KPC: *Klebsiella pneumoniae* Carbapenemase
HGT: Horizontal gene transfer
EMB: Eosin methylene blue
CLSI: Clinical and Laboratory Standards Institute
CDT: Combined disc test
DDST: Double disc synergy test
mCIM: Modified carbapenem inactivation method
eCIM: EDTA-CIM
PBA: Phenylboronic acid
DMSO: Dimethyl sulfoxide (DMSO)
EIEC: Enteroinvasive *E. coli*
NDM: New Delhi metallo-beta-lactamase
IMP: Imipenemase
VIM: Verona integron-encoded metallo-beta-lactamase
OXA: Oxacillinases
ESBL: Extended-spectrum β-lactamases
SIM: Sulfur indole motility
MR/VP: Methyl red/Voges-Proskauer
